# Periodontal status and oral hygiene practices among adults in a peri-urban fishing community in Ghana

**DOI:** 10.11604/pamj.2022.42.126.24557

**Published:** 2022-06-16

**Authors:** Daniel Tormeti, Harold Nii-Aponsah, Josephine Sackeyfio, Paa Kwesi Blankson, Neil Quartey-Papafio, Michael Arthur, Tom Akuetteh Ndanu

**Affiliations:** 1 Department of Community and Preventive Dentistry, Dental School, University of Ghana, Accra, Ghana,; 2 School of Health Studies, College of Health and Human Sciences, Northern Illinois University, Illinois, USA,; 3 Department of Oral and Maxillofacial Surgery, Korle-Bu Teaching Hospital, Accra, Ghana,; 4 Department of Biomaterials Science, Dental School, University of Ghana, Accra, Ghana,; 5 Dental Unit, Ghana Police Hospital, Accra, Ghana

**Keywords:** Periodontitis, gingivitis, oral hygiene, plaque, fisherfolk, Ghana

## Abstract

**Introduction:**

fisherfolk play a major role in emerging economies such as Ghana. While many fishing communities are noted to be underdeveloped, fisherfolk are considered to neglect their oral hygiene, while being prone to certain conditions due to peculiar risks. The purpose of this study was to determine the periodontal health status of adults in a selected fishing community in Ghana.

**Methods:**

a descriptive cross-sectional study was carried out to assess the periodontal status of adults in Jamestown, a peri-urban area in Ghana. Data acquisition was by means of a structured questionnaire and periodontal examination. Information obtained consisted of demographic data (age, sex, education and occupation category) oral hygiene practices (type of teeth cleaning materials, methods of tooth cleansing, frequency of dental visits and reasons for the visit) and periodontal clinical parameters (plaque index, and community periodontal index of treatment needs).

**Results:**

a total of 276 participants were included in the study, with ages ranging from 21 to 70 years. The participants were made up of 138 fisherfolk and 138 non-fisherfolk. Males had worse scores for periodontal disease compared to females. Plaque score did not vary among age groups, but changed significantly between educational level and occupational categories. CPITN varied significantly between educational levels, age categories and occupational categories.

**Conclusion:**

the study found inhabitants of the fishing community of Jamestown to have a generally unsatisfactory periodontal status, but worse for the fisherfolk in the community.

## Introduction

Periodontal disease, including gingivitis and periodontitis, is a leading cause of tooth loss in adults globally with a reported prevalence varying between 10-60% [1]. Gingivitis is a reversible form of periodontal disease, in which inflammation is confined to the gingiva without destruction of the supporting tissues. Periodontitis on the other hand, is the irreversible destruction of the deeper structures of the periodontium with resultant connective tissue attachment and alveolar bone loss, periodontal pocket formation, tooth mobility and eventual tooth loss [2]. Several factors are implicated in the etiology of periodontal disease, which range from local factors to systemic factors. Bacterial plaque, which arises as a result of poor dental hygiene has however been identified as the primary cause of periodontal disease [3]. Studies in different parts of the world demonstrate a direct correlation between the amount of bacterial deposit as measured by the Plaque Index, and the severity of gingival inflammation [4]. Several other factors may affect an individual´s oral hygiene practices such as age, gender, education, level of awareness and socioeconomic status [5]. Fishing serves as a major source of food and employment in West Africa [6]. Ghana´s fishing sector is a major contributor to its food security, accounting for about 75% of its total daily animal proteins in food, contributing to about 4.5% of gross domestic product (GDP), and providing employment for as many as 2.6 million Ghanaians [7]. It is reported that most of the people in fishing communities have minimal education, low income and are unaware of the effects of risk factors such as tobacco and alcohol use on oral health [8]. Some authors have also suggested that rural fisherfolk are prone to excess ultraviolet radiation due to constant exposure to the sun which makes them susceptible to develop skin and lip cancers [9]. With a gradually improving oral health care system in Ghana, it is important that this essential population, is not left out in policy implementations and public health benefits. There is also no description yet, of the oral health status of the fisherfolk in Ghana. This study therefore set out to explore the periodontal health status of adults in a selected fishing community in Accra.

## Methods

**Study design and setting:** this was a descriptive cross-sectional study carried out to assess the periodontal status of adults in Jamestown, a peri-urban area in Ghana. Jamestown is a fishing community located in the southern part of Accra, with population being largely of low socio-economic strata.

**Study population and eligibility criteria:** the study population included adults, 20 years and above, who were inhabitants of the area, and consented to be part of the study. Persons who were ill and could not be interviewed were excluded from the study. Individuals with less than 20 teeth were also omitted.

**Sampling method:** with a calculated sample size of 276, participants of the study were selected consecutively from community members who partook in a two-day outreach program organized by the Department of Community and Preventive Dentistry of the University of Ghana Dental School. All community members received oral hygiene education and were screened for common oral health diseases. People with oral health diseases were referred to a nearby Dental clinic for further management. Individuals meeting the inclusion criteria were then recruited to be part of the study.

**Data collection and variables:** data acquisition was by means of a structured questionnaire, and periodontal examination. Information obtained consisted of demographic data (age, sex, education and occupation category) oral hygiene practices (type of teeth cleaning materials, methods of tooth cleansing, frequency of dental visits and reasons for the visit) and periodontal clinical parameters (plaque index [10], and community periodontal index of treatment needs [11]). Plaque index was determined on six index teeth (16, 12, 24, 36,32 and 44). Each of the four surfaces of the teeth (buccal, lingual, mesial and distal) was given a score from 0-3: 0 being no plaque at all, 1 being a clean surface, but a thin film material can be removed from the gingival third by a probe, 2 being the presence of visible plaque, and 3 being where the tooth is covered with abundant plaque. The scores from the four areas of the teeth were added and divided by four (4) to obtain the plaque index for the tooth. The plaque index of the patient was obtained by summing the indices for all the six teeth and dividing by six. Community Periodontal Index of Treatment Needs (CPITN) was used to evaluate their periodontal health and oral hygiene status [12]. The CPITN periodontal probe was used to measure pocket depth, and teeth numbers 16, 11, 26, 36, 41 and 46 were used as index teeth for the CPI readings. The readings were based on codes 0,1,2,3,4 which correlates to the presence of healthy gingiva, no periodontal disease, bleeding on probing, calculus with plaque or a probing depth greater than 3 mm, a pocket depth of 4-5 mm and pocket depth of 6 mm or more respectively (Table 1). The Plaque index and the CPITN were further classified into the presence or absence of disease, where a value greater than 1 was categorized as the presence of disease.

**Table 1:** basic periodontal examination

CPITN code	Description
**0**	No disease (gingival pockets < 3 mm)
**1**	Bleeding on probing, but gingival pockets < 3 mm
**2**	Periodontal pocketing < 3 mm, but calculus present with or without plaque retentive factors such as “overhanging” restorations
**3**	Periodontal pockets 4 - 5 mm
**4**	Deep periodontal pockets > 6 mm

**Data analysis:** data were entered, processed and analyzed using Stata 14 software (StataCorp. College Station, TX). Background characteristics for all respondents were described, and descriptive summaries for all variables were reported. Tests of associations were done among the obtained variables and reported.

**Ethical considerations:** ethical approval was obtained from the University of Ghana School of Medicine and Dentistry Ethical Review Committee (CPDD/005/06/2018). Written and verbal consent were sought from all participants.

## Results

A total of 276 participants were included in the study, with ages ranging from 21 to 70 years. The participants were made up of 138 fisherfolk and 138 non-fisherfolk. Majority of both fisherfolk and non-fishing folk were aged between 31-40 years. Most of the respondents in the study area had been formally educated up to the primary level (44.6%), while 36.2% of them had not had any formal education. Furthermore, none of the participants had been educated to the tertiary level. Other background characteristics are shown in Table 2. Many of the respondents use only chewing sponge or stick alone for teeth brushing (44.9%), while 8% used toothbrush and paste alone. Some 47.1% used a combination in their oral hygiene care. Also, 34.8% of fishermen cleansed their teeth twice daily, compared to the 89.9% of non-fisherfolk. Seventy-five percent of the study population had never visited a dentist, while for the remaining who attended the dental clinic, major reasons were toothache (90%) (Table 3). Table 4 and Table 5 show the Plaque score and CIPTN distribution within the study population. There were significant changes in plaque score as well as CPITN among the sexes within the population. Males seemed to have had a worse score of periodontal disease compared to females (p<0.001). Plaque score did not vary among age categories, but changed significantly between educational level and the occupational categories (Table 4). However, CPITN varied significantly between educational levels, age categories and occupational categories (Table 5).

**Table 2:** background characteristics of respondents

Background characteristics	Number	Proportion
**Sex**		
Male	151	54.7
Female	125	45.3
**Age category**		
21-30	46	16.7
31-40	130	47.1
41-50	62	22.5
51-60	34	12.3
61-70	4	1.5
**Education**		
No formal education	100	36.2
Primary	123	44.6
JSS/middle School	41	14.9
Secondary	4	1.5
Technical/vocational	8	2.9
Tertiary	0	0.0
**Occupation status**		
Fisherfolk	138	50.0
Non-fisherfolk	138	50.0

**Table 3:** oral health practices and some oral health indicators

Responses	Number	Percent
**Method of cleaning the mouth**		
Toothbrush with tooth paste	22	8.0
Chewing stick/sponge	124	44.9
Combination of both	130	47.1
**Taught how to clean the mouth**		
**Yes**	274	99.3
**No**	2	0.7
**Ever visited the dental clinic**		
Yes	69	25.0
No	207	75.0
**Reason for dental visit**		
Toothache	62	90.0
Bleeding gums	7	10.0
Routine visit	0	0
**Bleed when brushing**		
Yes	248	89.9
No	28	10.1
**Experience of gum swelling**		
Yes	248	89.9
No	28	10.1

**Table 4:** factors associated with the plaque score

Variable	Plaque score			χ^2^ value! (Presence of disease)	P-value
	1	2	3		
**Sex**					<0.001*
Male	2	11	138	28.546	
Female	14	111	0		
**Age category**					0.274
21-30	3	31	12		
31-40	4	37	89	4.331	
41-50	6	30	26		
51-60	3	20	11		
61-70	0	4	0		
**Education**				29.631	<0.001*
No formal education	0	11	89		
Primary	6	68	49		
JSS/middle school	6	35	0		
Secondary	1	3	0		
Technical/vocational	3	5	0		
Tertiary	0	0	0		
**Occupation status**				16.985	<0.001*
Fisherfolk	0	0	138		
Non-fisherfolk	16	122	0		

!Fisher's test used where applicable significant values *Statistically

**Table 5:** factors associated with the CPITN score

Variable	CPITN				χ^2^ value! (presence of disease)	P-value
	1	2	3	4		
**Sex**					30.303	<0.001*
Male	4	9	50	88		
Female	31	91	3	0		
**Age category**					18.374	0.001*
21-30	13	18	8	7		
31-40	7	34	34	55		
41-50	11	25	6	20		
51-60	4	19	5	6		
61-70	0	4	0	0		
**Education**					24.898	<0.001*
No formal education	3	7	33	57		
Primary	17	57	18	31		
JSS/middle school	9	31	1	0		
Secondary	2	2	0	0		
Technical/vocational	4	3	1	0		
Tertiary	0	0	0	0		
**Occupation status**					40.083	<0.001*
Fisherfolk	0	0	50	88		
Non-fisherfolk	35	100	3	0		

!Fisher's test used where applicable *Statistically significant values

## Discussion

With the aim of giving some insight into the level of oral health care in similar communities, this study sought to assess the periodontal status of habitants of a fishing community in Accra. Generally, there was poor periodontal health, while this was found to vary significantly with different population characteristics. This study found males to have worse periodontal status, compared to females. This finding was consistent with a study done by Desvarieux *et al*. which similarly demonstrated that males have worse periodontal status and higher plaque scores than women [13]. Levin *et al*. also reported that men have poor periodontal status compared to women [14]. However, increased gingivitis was seen in women in a fishing community in Brazil, although it was found that calculus was increased among males in the same population [15]. It was also observed in this study, that members of this community were generally of a poor economic status, with 36% of them not having been formally educated at all. Several authors have opined, as suggested in this study, that formal education could have a direct effect on the oral health of the citizenry [16,17]. Paulander *et al*. also concluded that people with very low educational levels had relatively higher CPITN scores and poor plaque index than educated people in the same communities in all the ages [16]. This assertion creates a window of opportunity for oral health promotion interventions to utilize the formal educational system, as it could prove beneficial in effecting desired change in the long term.

We found that people of the community generally had high plaque index, with just 5.8% of the total respondents having an acceptable score. All fisherfolk, representing 50% of the sampled population, had a poor plaque index. This was consistent with findings of De Mesquita *et al*. who reported high plaque index among the adults of a fishing community with calculus presence of about 82% [15]. Like the Plaque score, CPITN values for this study were generally high. Thirty-six percent of the sampled population had visible calculus with plaque, and 31.9% had probing pocket depth of 6 mm or more. Furthermore, this study found that the fisherfolk had very poor CPITN scores compared to non-fisherfolk. Similar findings were reported by Asawa *et al*. (2014) in India, where fisherfolk demonstrated significant calculus presence and probing depth compared to non-fishermen [17]. While the Dentist population of Ghana could be improved upon, there has been substantial improvement over the past years to improve the distribution of healthcare facilities, as well as the availability of professionals. Furthermore, some basic oral services are currently included in the National Health Insurance scheme (NHIS) operated in Ghana. While this aims to ensure equitable access and financial coverage for basic health care, utilization by the communities with similar characteristics as in this study leaves much to be desired. Education is therefore essential to effect positive or desired health behaviour changes among community members, and to improve on utilization of the available resources [18].

Community medical outreaches have been important tools for the Government of Ghana, it´s agencies, and other non-governmental organisations. These initiatives are even more crucial, as certain rural areas may, in the short term, lack basic amenities and resources for the establishment of dental clinics. Dental outreaches should therefore be harnessed to deliver essential oral care to such deprived communities. This study was limited by the few variables collected in assessing the oral health practices, attitudes and characteristics of the population. The study design also limits the ability to generalize findings to the entire population, while the depth of exploration could have been improved by some qualitative elements. This study however provides a description of the periodontal state of a typical fishing community in Ghana, highlighting its peculiar characteristics and associations, which could be useful in informing community oral health strategies.

## Conclusion

The study found inhabitants of the fishing community of Jamestown, Ghana, to have a generally unsatisfactory periodontal status, but worse for the fisherfolk in the community.

### What is known about this topic

- *Periodontal disease, including gingivitis and periodontitis, is a leading cause of tooth loss in adults;*
- *Several factors are implicated in the etiology of periodontal disease, which include local factors and systemic factors, as well as social factors*.

### What this study adds

- *The characteristics of fishing communities in Ghana could predispose fisherfolk to poor health and periodontal diseases*.
